# Mycobacterium Bovis Spondylodiscitis: A Rare Complication of Intravesical Bacillus Calmette-Guérin Therapy

**DOI:** 10.5334/jbsr.2626

**Published:** 2022-07-06

**Authors:** Frédéric Quatannens, Peter Matthys, Charles-Edouard Heylen

**Affiliations:** 1 KU Leuven, BE; 2 Sint-Elisabeth, BE

**Keywords:** mycobacterium bovis, spondylodiscitis, BCG therapy

## Abstract

**Teaching point:** Mycobacterium bovis spondylodiscitis is a rare, often delayed, complication of intravesical Bacillus Calmette-Guérin therapy for bladder cancer.

## Case History

A 75-year-old patient with a history of high-grade urothelial cell carcinoma (UCC) treated with transurethral resection of bladder tumour (TURBT) and adjuvant instillation of Bacillus Calmette -Guérin (BCG) presented to the neurology ward with long-standing back pain irradiating to the lower limbs.

**Figure 1:**
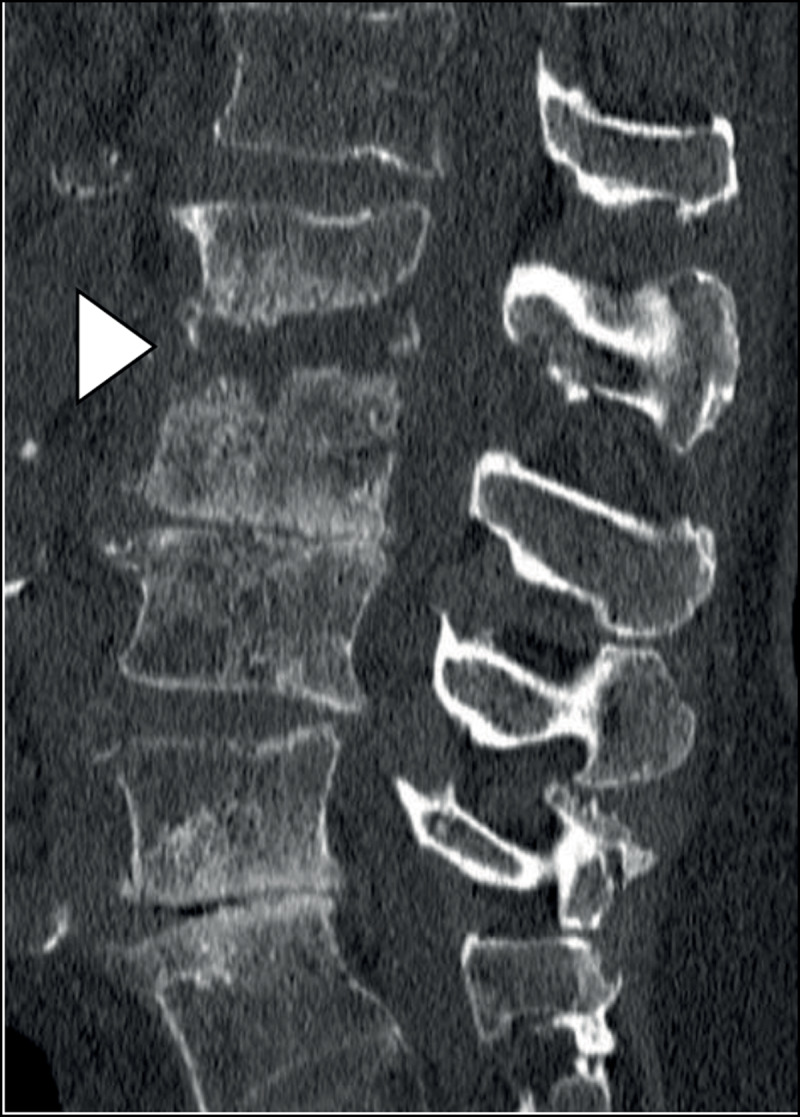


**Figure 2:**
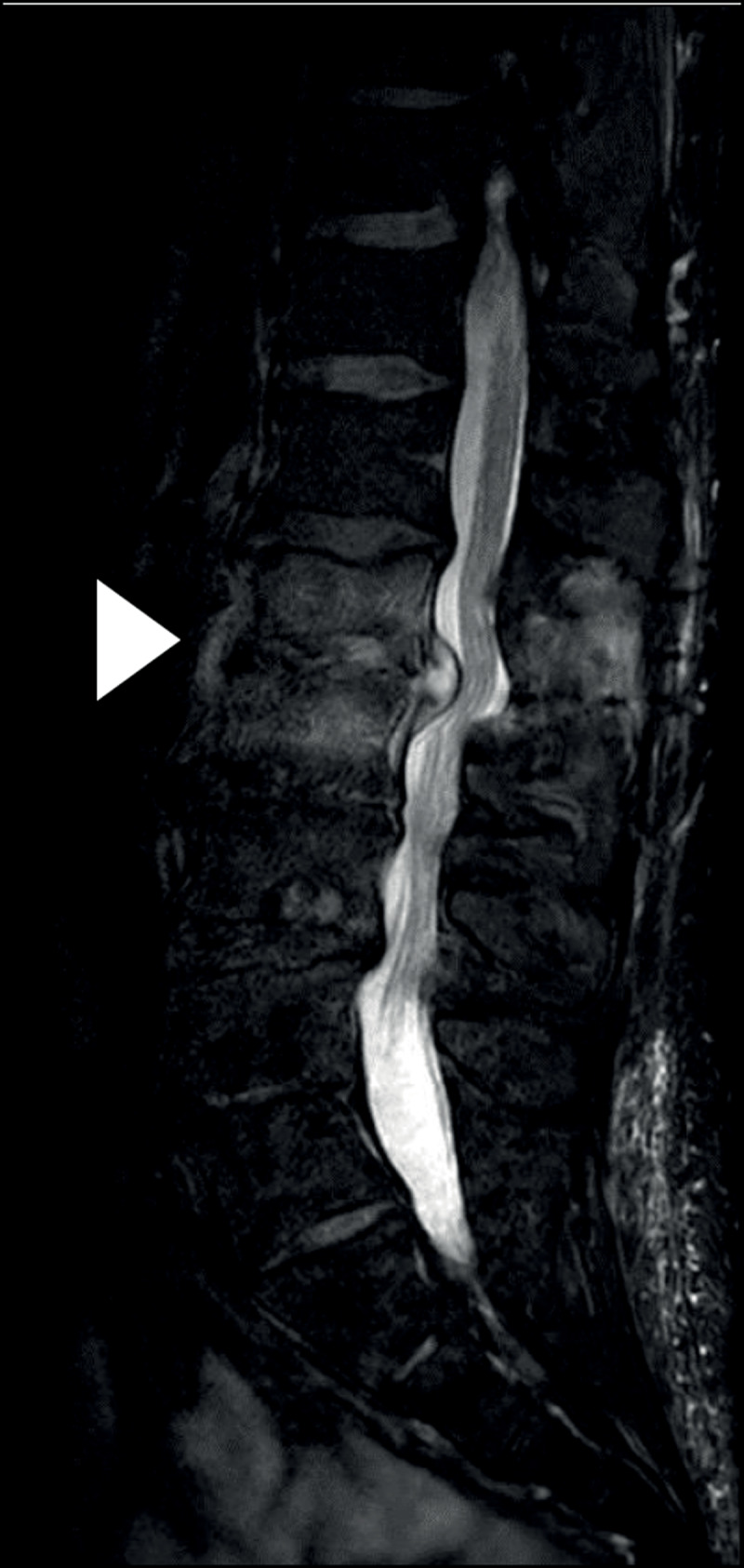


**Figure 3:**
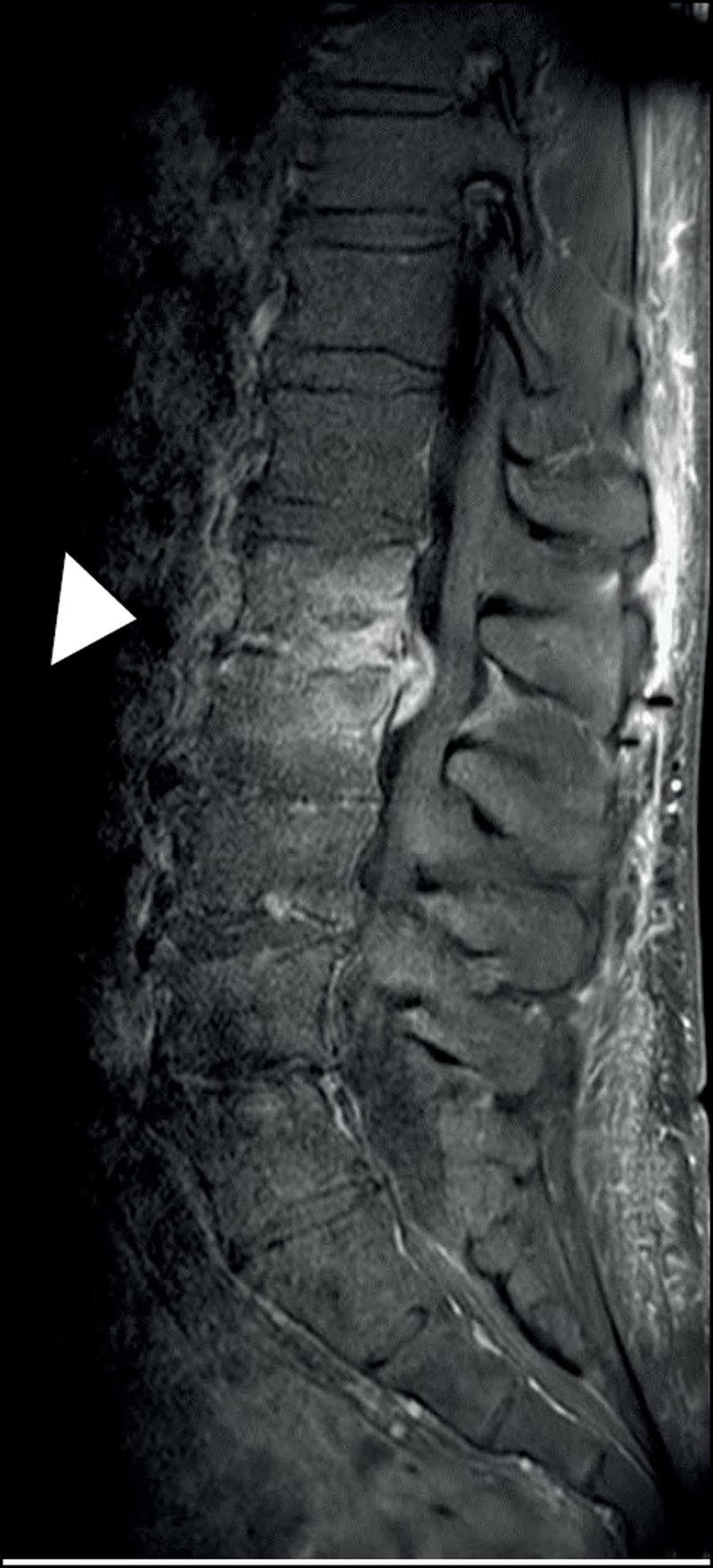


Computed tomography (CT) of the lumbar spine demonstrated narrowing of the L1–L2 intervertebral disc space, irregular end plates with erosions and loss of height of the vertebral bodies of L1 and L2 (arrowhead, ***Figure 1***), in comparison with the levels L2–L3 and L4–L5 which show narrowing of the intervertebral disc space with degenerative endplate changes without erosions, a typical image of osteo-arthritis. Magnetic resonance imaging (MRI) confirmed narrowing of the intervertebral disc space in the sagittal plane, with hyperintense signal of the vertebral bodies, the disc and the surrounding tissues on T2-weighted fat saturated images, consistent with oedema (arrowhead, ***Figure 2***). Contrast-enhanced T1-weighted images showed an important contrast enhancement of the disc and the surrounding soft tissues (arrowhead, ***Figure 3***).

The patient underwent lumbar spine decompression surgery during which a pus collection was found, drained and taken for culture. The patient then started an anti-tuberculous treatment consisting of rifampicin, isoniazid, ethambutol and pyrazinamide. Pyrazinamide was later stopped when Mycobacterium bovis Bacillus-Calmette Guérin (BCG) was identified on culture. The patient presented a good clinical evolution.

## Commentary

One of the rarer complications of intravesical BCG therapy is distant infection by mycobacterium bovis, which includes spondylodiscitis. Symptoms are identical to that of tuberculous spondylodiscitis (Pott’s disease), including back pain, leg weakness, fever, weight loss and paresthesias [1]. CT and MRI show endplate destruction of contiguous vertebral bodies. Hyperkyphosis may be seen due to the vertebral body collapse. Marrow infiltration, edema and enhancement are better seen on MRI, which also shows better evaluation of the spinal canal for possible epidural abscess or cord compression. Spreading into the surrounding soft tissues is common, mostly through the longitudinal ligaments. Involvement of the psoas muscles or aorta can be seen. BCG spondylodiscitis is treated by antimycobacterial therapy and in severe cases, surgical interventions such as spinal stabilization, abscess drainage or spinal cord decompression are required [1].
